# Left inferior epigastric artery injury in COVID-19 patient. Case report and literature review

**DOI:** 10.1016/j.ijscr.2020.09.198

**Published:** 2020-10-04

**Authors:** Ifrat Bakirov, Gultakin Bakirova, Yousef Albalawi, Ayed Y. Asiri, Fahad Faqihi, Ilkin Bakirli, Dema Hammami, Hasan Bakirli, Najmeddin M. Mandow

**Affiliations:** a Imam Abdulrahman Alfaisal Hospital, Riyadh, Saudi Arabia; b King Saud Medical City, Riyadh, Saudi Arabia; c Slovak Medical University, Bratislava, Slovakia; d National Institute of Cardiovascular Diseases, Bratislava, Slovakia; e Cyril and Methodius Hospital, Bratislava, Slovakia

**Keywords:** COVID-19, Inferior epigastric artery, Rectus sheath haematoma, Computed tomography, Laparotomy, Case report

## Abstract

**Introduction:**

Management of COVID-19 pneumonia cases is a medical challenge. However, the situation becomes worse if the patient has coexisting morbidities or newly developed complications. The study is about managing rectus sheath haematoma (RSH) in a patient with COVID-19 pneumonia.

**Presentation of case:**

The patient was a 75-year-old male, presenting with bilateral COVID-19 pneumonia, with pulmonary embolism complications. Therapeutic anticoagulation by subcutaneous Clexane injection was administered. A left rectus haematoma was observed, and the patient fell and underwent haemorrhagic shock. Laparotomy was done for the evacuation of the haematoma.

**Discussion:**

Contrast-enhanced computed tomography (CECT) is an essential tool for diagnosing RSH, identifying the source of bleeding, type of haematoma, and compression of the urinary system.

**Conclusion:**

Surgical management of RSH in COVID-19 patients is superior to interventional radiology during the rush pandemic period.

## Highlights

• Rectus sheath haematoma is a relatively rare condition.
• Rectus sheath haematoma in COVID-19 patients.
• Lower Paramedian incision - rapid evacuation of the rectus sheath haematoma.

## 1 Introduction

The current case study is about managing rectus sheath haematoma (RSH) in a patient with COVID-19 pneumonia. Inferior Epigastric Artery Injury (IEAI) caused by blunt abdominal trauma is rare, but regardless of the cause, a potentially life-threatening condition [1]. IEAI commonly occurs due to iatrogenic injuries during surgery or procedure (laparoscopy, paracentesis, percutaneous drain) [[1], [2], [3], [4], [5]].

The risk factors of RSH are anticoagulation therapy, hypertension, atherosclerosis, and chronic cough, with pregnancy and old age, also in the list [[6], [7], [8], [9]]. By CT appearances, three types of haematoma can be distinguished [[4], [5], [6], [7], [8]].

## 2 Presentation of case

**Fig. 1:**
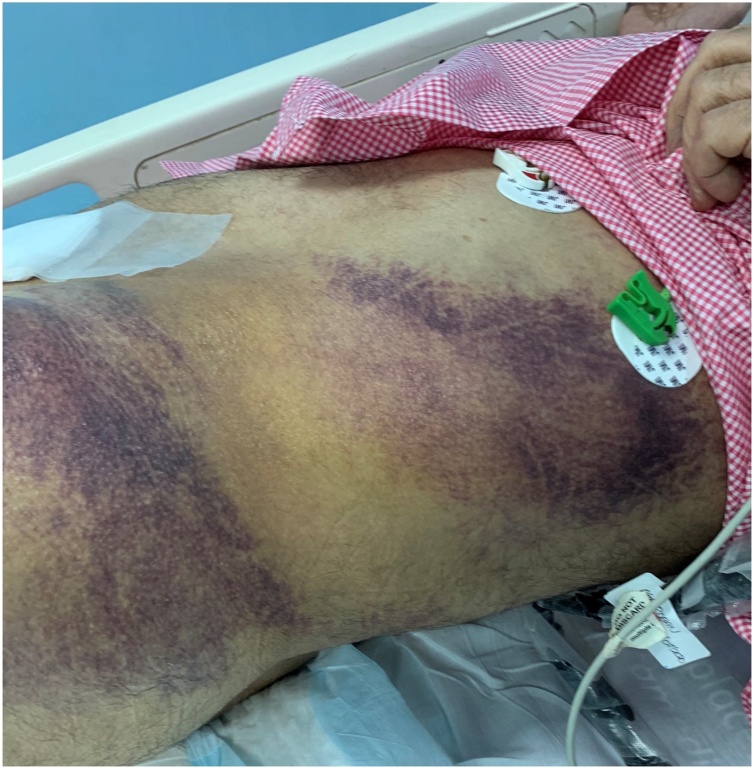
Large ecchymosis on the left trunk and hip. Skin discolorations are still present 10 days after the trauma.

A 75-year-old male patient having mild hypertension treatment presented with complaints of fever, cough, and shortness of breath on 10th June 2020 during the COVID rush period in Riyadh city. The patient had already been tested positive for COVID-19 and was in self-quarantine but was hospitalized due to oxygen requirement. CECT of the chest showed bilateral opacifications, matching COVID-19 pneumonia and bilateral segmental and subsegmental PE. After being treated in the ICU for three days, including noninvasive ventilation and therapeutic anticoagulation by subcutaneous Clexane injection, the patient was shifted to a specialized COVID ward for further management. Unfortunately, in the ward after four days of admission, early morning, he collapsed suddenly while going to the bathroom. The patient was found unconscious, Glasgow Coma Scale (GCS) 8/15, Pulse (Ps) -120, BP - 80/60 mmHg. The patient was resuscitated, intubated, connected to a ventilator, and was given a 1.5 L bolus of Ringer Lactate solution. The lacerations found over the left eyebrow and the left side of the skull with mild oozing was sutured, and dressings were applied. Multiple abrasions, friction burns, bruises, contusions, and haematomas over the abdomen were also found, mainly on the left side at the paraumbilical and left loin areas (Fig. 1). Foley’s catheter was inserted, and mild haematuria was found. Anticoagulation was stopped.

**Fig. 2:**
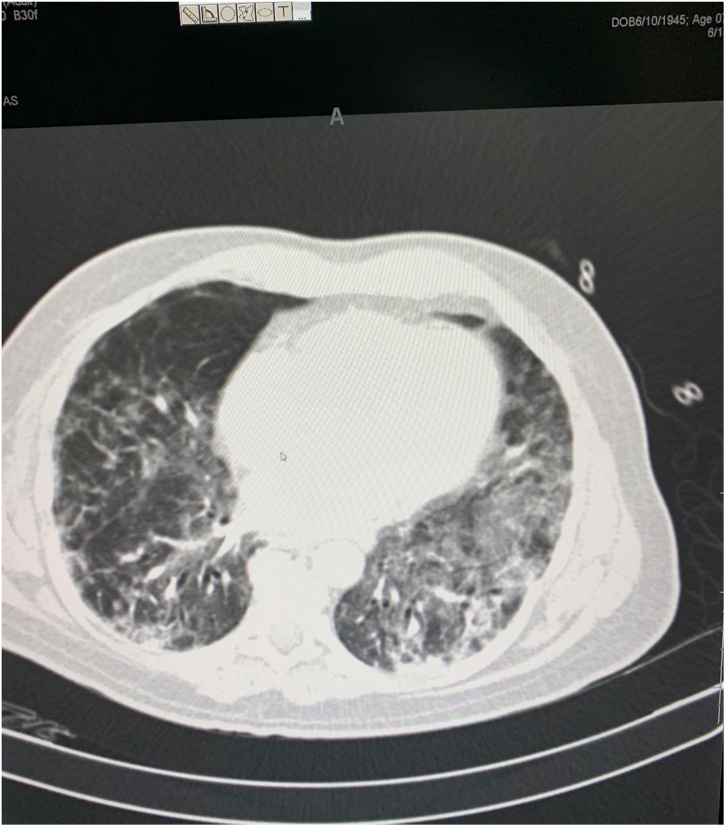
CT chest shows signs of bilateral COVID-19 pneumonia.

The patient was stabilized (Ps-90, BP- 120/70 mmHg) and shifted to the CT room for secondary evaluation. CECT study shows normal age-related findings in the brain, COVID-19 related changes in both lungs, bilateral, diffuse patchy peripheral, subpleural consolidated opacities, more evident at the lower lobes, associated with interlobar and intralobar septal thickening without any evidence of pleural effusion at each side. These features are suggestive of COVID-19 related pneumonia (Fig. 2).

Trauma-related findings noticed in the abdomen were a large left RSH extending along all length of muscle with a maximum width of 6 cm and a maximum depth of 4 cm, not crossing the midline but appears to be dissecting the transversalis fascia in its inferior part towards the left pubic bone and pelvis and shifting the urinary bladder towards the right side. A small amount of extraperitoneal free fluid was seen at the level of the anterior surface of the left psoas muscle. A grade 2 splenic injury, in the form of multiple small intraparenchymal haematomas, with no free fluid in the peritoneal cavity, was also seen. The nephrogram phase of both kidneys was normal. UB was empty but appeared to be compressed and revealed a right-side deviation by the lower part of haematoma. No evidence of free gas inside the abdomen.

The patient was re-shifted to the ICU and treated conservatively, given four units of grouped and matched packed red blood cells (RBC), four units of fresh frozen plasma (FFP), and four units of PLT mass transfused. Inotropic support was initiated, and BP maintained at 100/60 mmHg to have better control of haemorrhage.

**Fig. 3:**
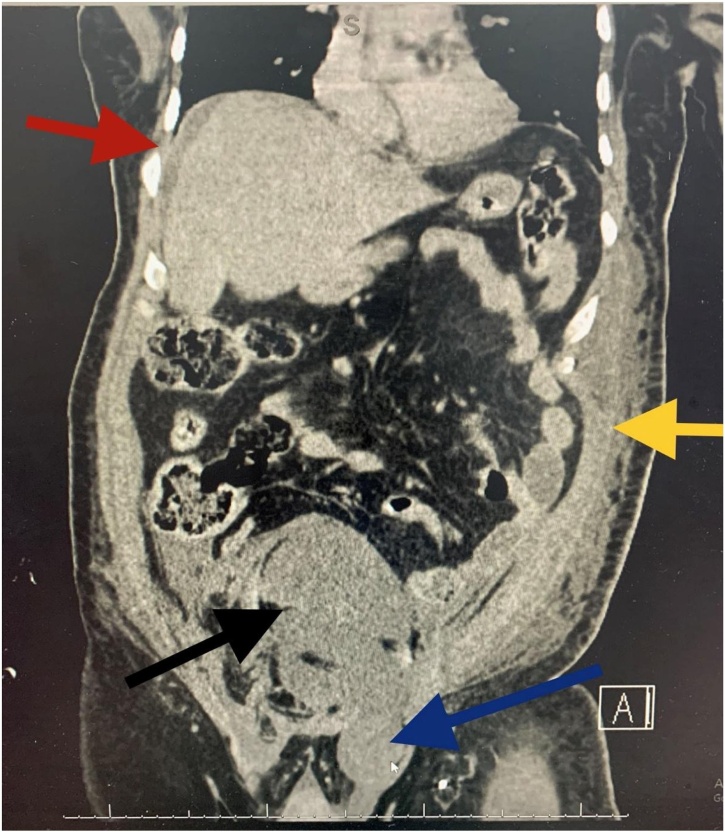
CECT coronal view. Black arrow showing the RSH dissected down to the pelvis, blue arrow showing inguinal extension, yellow arrow showing left abdominal wall haematoma and the red arrow showing mild perihepatic free fluid.

**Fig. 4:**
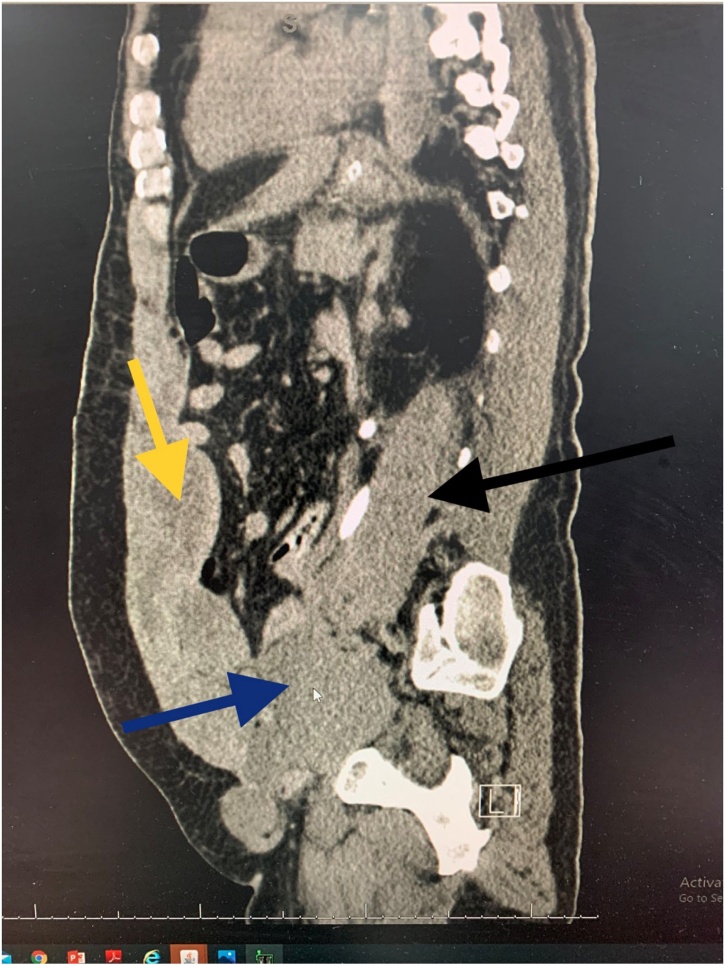
All 3 components of the haematoma: yellow arrow showing the RSH, blue arrow showing the pelvic extension and the black arrow showing the retroperitoneal part surrounding the left ureter.

**Fig. 5:**
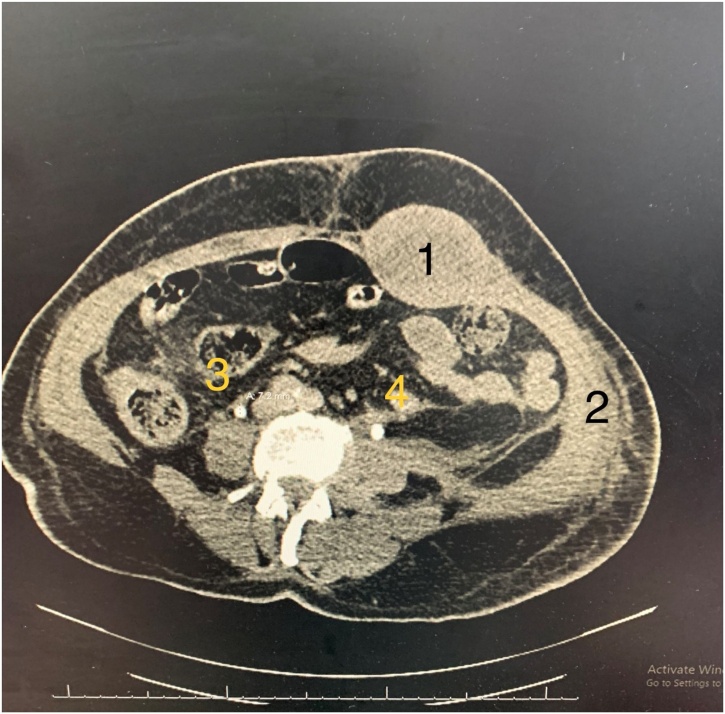
1-RSH, 2. Left abdominal wall haematoma, 3- mild dilated right ureter 7.5 mm, 4- dilated left ureter 10.5 mm.

**Fig. 6:**
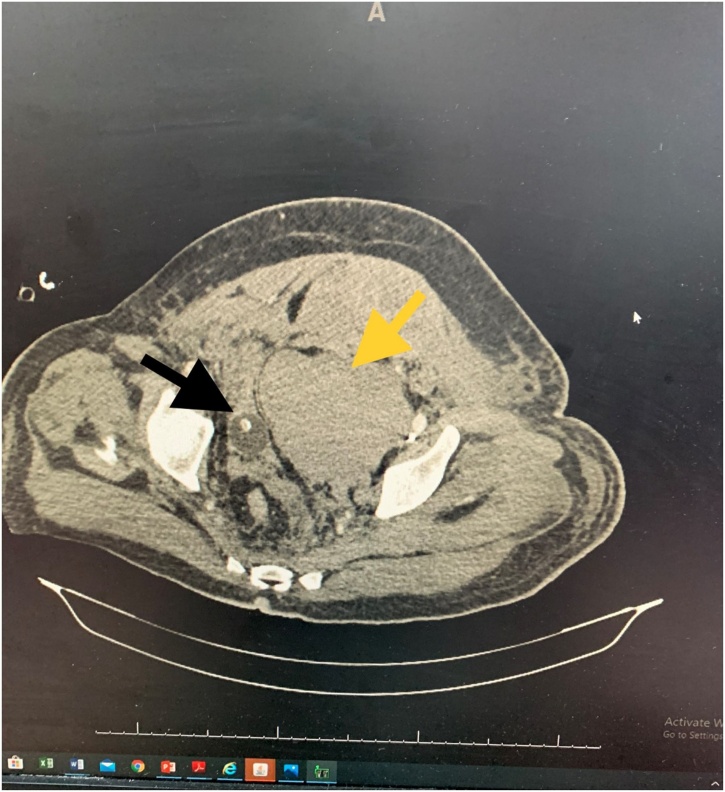
Yellow arrow showing the left RSH extended down to the pelvis and the black arrow showing the displaced UB towards the right side.

Despite adequate treatment, he was continuously deteriorating, white blood cells (WBC) jumped to 40,470 (18,700 before fall, 20,300 immediately after fall, and 37,100 12 h after fall), lactate level became 3.7 mmol/L 12 h after fall and 5.7 mmol/L 24 h after fall and renal function tests (S. urea 18 mmol/L, S. creatinine 352 mmol/L) was raised, the patient became anuric despite transfusions. Haemoglobin level again dropped to 7.6 g/dL (before trauma 12 g/dL, few hours after trauma 6.9, after four units of transfusion 9.8 g/dL). Laparotomy was decided after 24 h of ICU management, as the patient was not recovering from the shock, haemoglobin was not maintained, and laboratory investigations showed signs of progressive sepsis. The patient was given another two units of packed RBC and two units of FFP; the patient was then shifted to CECT again to have a preoperative assessment, to localize the constant bleeding source if possible, and to rule out active bleeding from the spleen. CECT found a marked increase in the size of the lower dissecting part of a previously diagnosed left-sided huge RSH, to become filling most of the left pelvis (Fig. 3), causing compressing and deviating toward the right side of UB. There was a newly appeared mild amount of free fluid around the liver and spleen. A similar appearance of minimal extraperitoneal free fluid at the psoas muscle (Fig. 4), with newly appeared minimal amount fluid at descending colon and left perinephric space (left retroperitoneal), was also seen. Both kidneys reveal normal nephrogram and excretory phases, and outlining of the ureters are mildly dilated (Fig. 5), seen 60 s after injection of contrast media, about 7.5 mm on the right side and 10.5 mm on the left side- most likely related to right side deviation of UB and compression by large pelvic haematoma (Fig. 6). Dilated outlining of the ureters continued till the distal end before their entrance to UB. No contrast reached to the UB. The same findings are seen related to the grade 2 splenic injury.

The CT reports revealed bleeding is continuous, the source is at the abdominal wall, not intraperitoneal, haematoma dissected down to the pelvis, and compression and deviation of UB caused a postrenal renal failure and anuria. Emergency surgery was decided, all operation staff and anaesthesia team were informed about the laparotomy in this COVID-19 positive case. The patient was shifted directly to the operating room (OR) without being in the preoperative hold area. All the involved personnel used airborne protective equipment. The left paramedian incision was done, the rectus sheath opened, and the free blood along with the large haematoma lying under the muscle was drained and evacuated. IEAI was found at the lower lateral part of the rectus sheath, ligated by silk 2.0, and bleeding was controlled. The pelvic and perivesical haematoma was evacuated. The posterior wall of the rectus sheath and peritoneum was opened, the left external iliac artery was exposed, no other source of bleeding was found, and moderate amounts of clots and free blood were removed from the rectovesical pouch. A drain was placed into the pelvis intraperitoneally and to the Reitzius space to drain the perivesical area.

**Fig. 7:**
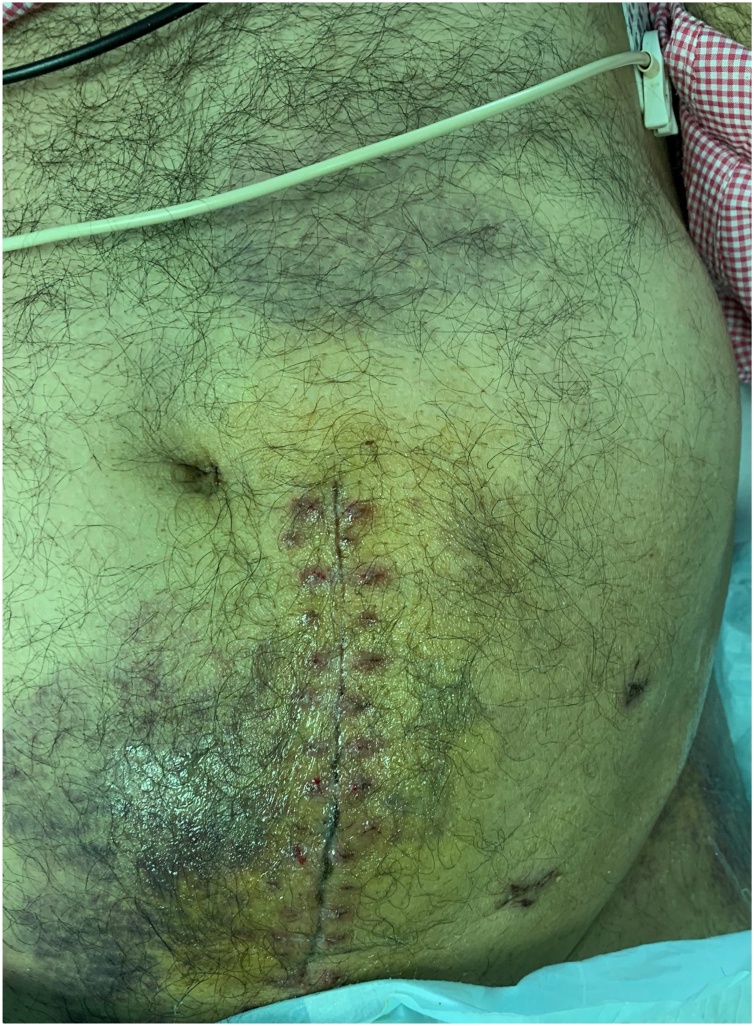
Post Laparotomy wound healed by primary intention. Ecchymosis at the surgery side (left) resolved faster than right side. 14th post-laparotomy day.

During surgery, after the evacuation of the haematoma and controlling the bleeding, BP raised, and 1 of 4 inotropes – adrenaline infusion was stopped. Postoperatively, the patient improved dramatically; 3 remaining inotropes -Noradrenaline, Vasopressin, and Dopamine, were withdrawn within 24 h, urine output normalised, and the patient was extubated. Kidney function tests normalised within two days: S. Urea 8 mmol/L, S. Creatinine 125 mmol/L. Forty-eight hours after laparotomy, a prophylactic dose of subcutaneous UFH injection was restarted. Therapeutic heparinization was regained on the 5th day postoperatively. The patient developed a new complication: heparin-induced thrombocytopenia (HIT), with platelet (PLT) count reduced to 67 000/μl. Heparin infusion was stopped, and argatroban infusion was started and then changed to apixaban tablets: the laparotomy wound and lacerations over the left eyebrow and left parietal area healed by primary intention. Face and skull stitches were removed on the 6th postoperative day. The laparotomy wound skin clips were removed 12–13 days after the surgery (Fig. 7). SARS-CoV-2 RT-PCR test results turned negative on the 13th postoperative day. The patient was discharged home in good condition with oral anticoagulation 14 days after the laparotomy.

## 3 Discussion

IEAI requires early diagnosis and timely intervention in order to achieve the desired outcome. Multiphase CT is the investigation modality of choice for finding the source of bleeding and differentiation of arterial vs. venous haemorrhage. In patients where CT angiography is contraindicated, Doppler ultrasound, and red blood cell scintigraphy [5,[9], [10], [11]] are used. Treatment of RSH is usually expectant and may include fluid resuscitation, blood transfusions, and management of pain. Uncommonly, intravascular embolization or surgery may be needed.

## 4 Conclusion

The surgical option opted as our aim was not only to control the haemorrhage, but also to evacuate the haematoma to release the urinary obstruction. After the evacuation of the haematoma, the BP of the patient immediately raised, possibly meaning that the haematoma was compressing the left external iliac vein as well.

## Declaration of Competing Interest

The authors report no declarations of interest.

## Funding

No funding sources.

## Ethical approval

This is only single case report and no Ethical Approval is required.

## Consent

Written informed consent is obtained from the patient for publishing an article and for photos. The copy of the consent is available for review by the Editor-in-Chief of this journal on request.

## Author contribution

All authors equally drafted and reviewed the manuscript. The final version of the manuscript was approved by all authors.

## Registration of research studies

N/A.

## Guarantor

Ifrat Isa Bakirov is the guarantor.

## Availability of data and materials

Datasets used and/or analysed during the current study are available from the corresponding author on reasonable request.
